# Denoising OCT videos based on temporal redundancy

**DOI:** 10.1038/s41598-024-56935-0

**Published:** 2024-03-19

**Authors:** Emmanuelle Richer, Marissé Masís Solano, Farida Cheriet, Mark R. Lesk, Santiago Costantino

**Affiliations:** 1 Department of Computer Engineering and Software Engineering, École Polytechnique de Montréal, Montreal, QC H3T 1J4 Canada; 2 Maisonneuve-Rosemont Hospital Research Center, Montreal, QC H1T 2M4 Canada; 3 Department of Ophthalmology, Université de Montréal, Montreal, QC H3T 1P1 Canada

**Keywords:** Translational research, Biomedical engineering, Computer science, Imaging techniques

## Abstract

The identification of eye diseases and their progression often relies on a clear visualization of the anatomy and on different metrics extracted from Optical Coherence Tomography (OCT) B-scans. However, speckle noise hinders the quality of rapid OCT imaging, hampering the extraction and reliability of biomarkers that require time series. By synchronizing the acquisition of OCT images with the timing of the cardiac pulse, we transform a low-quality OCT video into a clear version by phase-wrapping each frame to the heart pulsation and averaging frames that correspond to the same instant in the cardiac cycle. Here, we compare the performance of our one-cycle denoising strategy with a deep-learning architecture, Noise2Noise, as well as classical denoising methods such as BM3D and Non-Local Means (NLM). We systematically analyze different image quality descriptors as well as region-specific metrics to assess the denoising performance based on the anatomy of the eye. The one-cycle method achieves the highest denoising performance, increases image quality and preserves the high-resolution structures within the eye tissues. The proposed workflow can be readily implemented in a clinical setting.

## Introduction

Technological advances in OCT imaging have allowed unprecedented resolution and signal-to-noise ratio for the visualization of detailed anatomical structures in the eye that are relevant to diagnosis. Single B-scans, 3-dimensional volume reconstructions and even the vasculature are routinely used to extract metrics that allow clinicians to identify oculopathies and monitor their progression. In the case of glaucoma, such quantifiable biomarkers include retinal nerve fiber layer (RNFL) thickness, cup-to-disk ratio, neuroretinal rim area, disk area, and ganglion cell layer thickness, to quantify disease stage and progression^1,2^. Much of current glaucoma research is focused on the mechanical properties of the eye, which can be extracted from the dynamics of ocular tissue. Hence the need for reliable methods that preserve the high quality of time series images obtained with a short integration time compatible with the clinical setting.

Intraocular pressure (IOP) is the only actionable risk factor for glaucoma patients. Treatments, whether drugs, laser, or surgery, all aim to decrease pressure to limit the progression of the disease^2^. However, not all glaucoma subjects have elevated IOP and increased IOP is not necessarily an indicator of glaucomatous damage^3^. Furthermore, the mechanisms and pathogenesis of glaucoma are still unclear^2^ and research aims to find other biomarkers, straying away from IOP-based descriptors. The characterization of ocular biomechanics, and particularly the calculation of stress and strain in retinal tissues driven by several stimuli was characterized in numerical simulations based on finite element modeling^4–6^, or in non-human primates subjected to acutely elevated IOP^7–9^. Human subjects with high and normal-tension glaucoma were studied using a digital volume correlation technique to map deformations^10^, a 3D tracking algorithm based on optimizing numerical transformations of OCT volumes was used to quantify strains in optic nerves of glaucoma subjects after IOP reduction by trabeculectomy^11^, and displacement maps were obtained to characterize the behavior of the retina and optic nerve head in normal and myopia cohorts subjected to optic nerve physiological traction due to eye movement^12^.

The cardiac and ocular systems are closely intertwined, and many believe that one system can bring information to the other^13,14^, for example by imaging retinal structures to enhance cardiovascular pathologies detection protocols^15–18^. OCT angiography utilizes the variation of the OCT signal taken at the same cross-section of the eye to visualize ocular vasculature^19^. This visualization helps clinicians in identifying several pathologies, from age related macular degeneration to diabetic retinopathy and more. Doppler OCT enables the visualization and quantification of retinal blood flow, in addition to the structural anatomy given by OCT^20^. Another study used a pulse oximeter to monitor retinal blood flow using OCT imaging^21^. A second group, using a methodology similar to the one proposed here, used the cardiac signal of an additional pulse oximeter to improve the sampling of pulsatile blood flow using lower frame-rate systems in Doppler OCT applications^22^.

Advances in OCT engineering have enabled faster frame rates and larger fields of view, enabling the performance of dynamic imaging of ocular structures, such as the retina, optic nerve head (ONH) and the lamina cribrosa. Dynamic imaging and analysis help to understand physiological changes due to pulsatile blood flow deformations and pulsatile IOP changes^23^. However, speckle noise significantly hinders the quality of these images. Biomarkers computed on the scans themselves, i.e., RNFL thickness, cup-to-disk ratio, and even new approaches that quantify local strain, are all dependent on signal quality and are severely affected by noise. Averaging successive B-scans, either temporally or spatially, can remove the noise naturally present in OCT images and is the most efficient noise removal technique used to this day in clinical devices^24^. Yet, this averaging cannot be applied naively to suppress noise in movies, as it erases the small variations due to tissue movement and temporal changes.

Several groups have focused on OCT denoising methods in recent years, most of them using new and improved deep learning architectures. The Noise2Noise architecture^25^ was first introduced for non-medical images and later applied to OCT scans^26^. The Noise2void architecture was proposed as a modification of Noise2Noise^27^, where the unsupervised training is done without the need for paired noisy images, instead using a blind-spot masking scheme with single noisy images. The latter model was applied to OCT scans as well^28^. Researchers proposed a conditional generative adversarial network to produce contrast-enhanced and speckle-reduced scans^29^. Another study put forward a convolutional neural network (CNN) trained to learn the difference between noisy OCT scans (with added Gaussian noise) and their corresponding multi-frame OCT scan^30^. Similarly, one group trained a CNN (a modified deep CNN) to denoise scans based on their multi-frame denoised versions^31^. This special architecture used a multi-scale structural similarity index as the loss function to obtain denoised images that preserve the structural information present in the images. Although all these methods have been applied to static OCT images or volumes, they could potentially be adapted to dynamic imaging.

Several drawbacks can be found in these methods: a large quantity of data is needed to train these networks without overfitting, they are complex and require significant resources (large amounts of memory, multiple high-performing GPUs), and they lack ground truth images for training supervised architectures. While the trained networks enable fast inference times, the main challenge of using deep learning methods, especially those using a generative workflow, is the lack of reliability of the resulting pixel intensity values. The black-box problem inherent in such models is well known and is especially important in biomedical imaging^32–35^. Measurements computed from the scans will influence diagnosis and treatment, and therefore the reliability of the pixel values in denoised scans is paramount.

Using the heart pulsation captured with a simple pulse oximeter synchronized to the OCT video acquisition, we propose instead a video denoising pipeline based on averaging images corresponding to the same structures taken at equivalent instants in the cardiac cycle. Our workflow (one-cycle video denoising) is particularly easy to implement in a clinical setting as it only requires a pulse sensor, and the computational workload is very light. Furthermore, all manipulations to the scans can be interpreted to support reliability of the numerical metrics extracted. The implementation details of our method are presented in the methods below and the code is made available in a public repository. We compare our denoising methods performance with four other denoising techniques: BM3D^36,37^, Non-Local Means (NLM)^38^, and a deep-learning architecture, Noise2Noise, trained in unsupervised and supervised ways. This architecture was selected for the comparison due to its well-established denoising capability and straight-forward implementation. The capability of neural networks to develop high complexity algorithms could allow the development of a single scan denoising scheme producing high quality images. Furthermore, we present a set of global and region-specific image quality metrics to estimate the denoising performance in terms of tissue anatomy in the retina and optic nerve head.

## Results

We used ten metrics (five global and five local, as described in Sections “Global image quality quantification”, “Region-specific image quality quantification” and “Movement preservation quantification” below) to assess the denoising performance and compare all methods both globally and in specific anatomically defined ROIs. The Wilcoxon test was applied between the one-cycle workflow metrics and every other method (including no denoising, i.e., the original B-scans). Every metric was computed on 100 different images from each video (i.e., each of the 21 scanned eyes). For our method, this was simply all the frames of the one-cycle video. For the other denoising methods, the 100 images per subject were randomly selected from the test sets.

**Figure 1:**
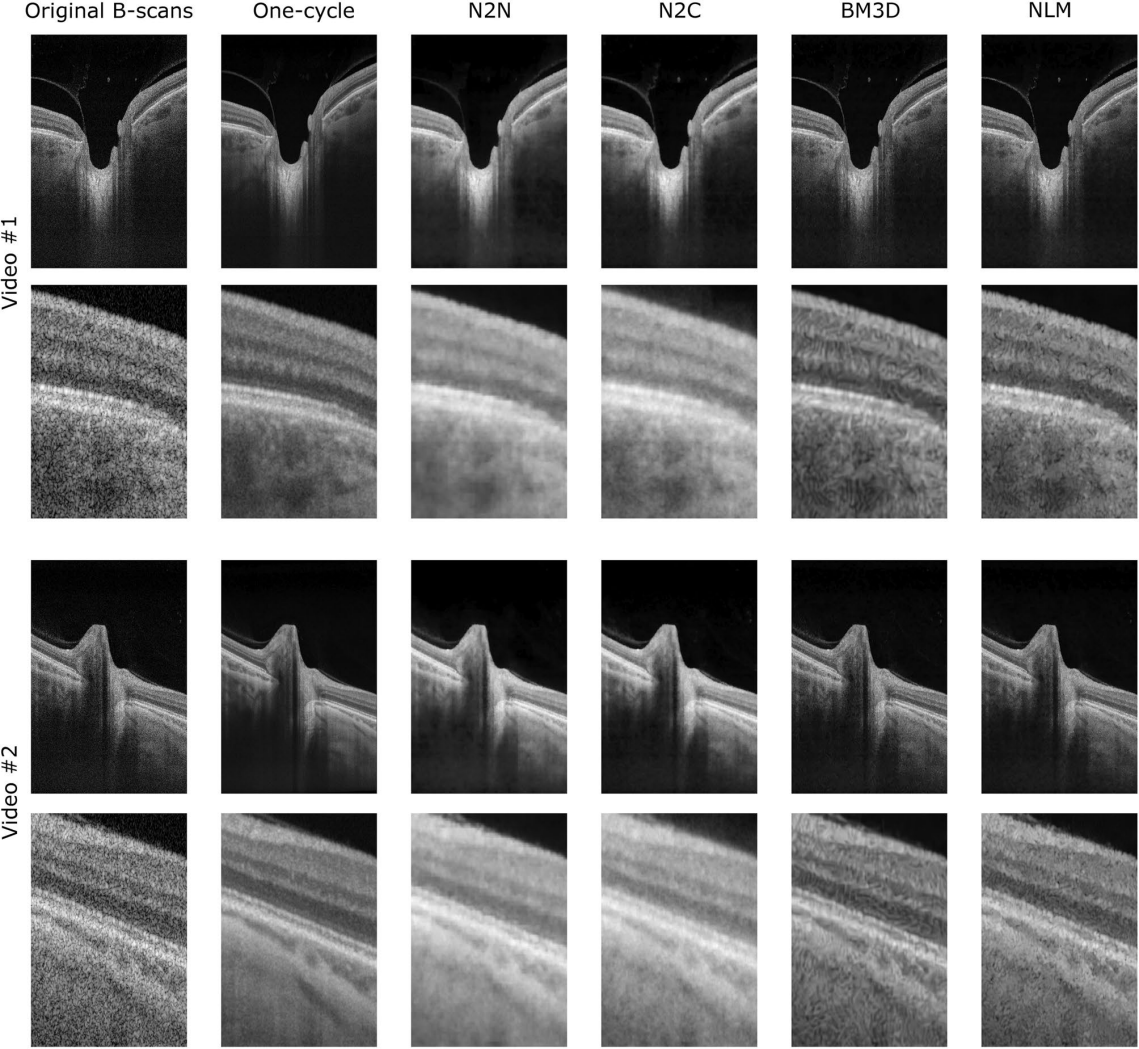
Visual comparison of the denoised scans with all five denoising workflows. Qualitative comparison between the original B-scans (left) and the denoised scans using the one-cycle workflow (second column), the Noise2Noise (N2N) model (third column), Noise2Clean (N2C) model (fourth column), BM3D (fifth column) and NLM (sixth column) on two different eyes (rows). Separate rows show the whole scan after denoising as well as a zoomed portion of the RNFL to RPE layers of the retina. These B-scans were not part of the training sets of the neural networks.

Figure 1 shows examples of the output of the algorithms applied to individual B-scans, while the whole movies for these same subjects can be found in the Supplementary material.

### Image quality metrics: all five methods show improved contrast and higher similarity between the scans

**Figure 2:**
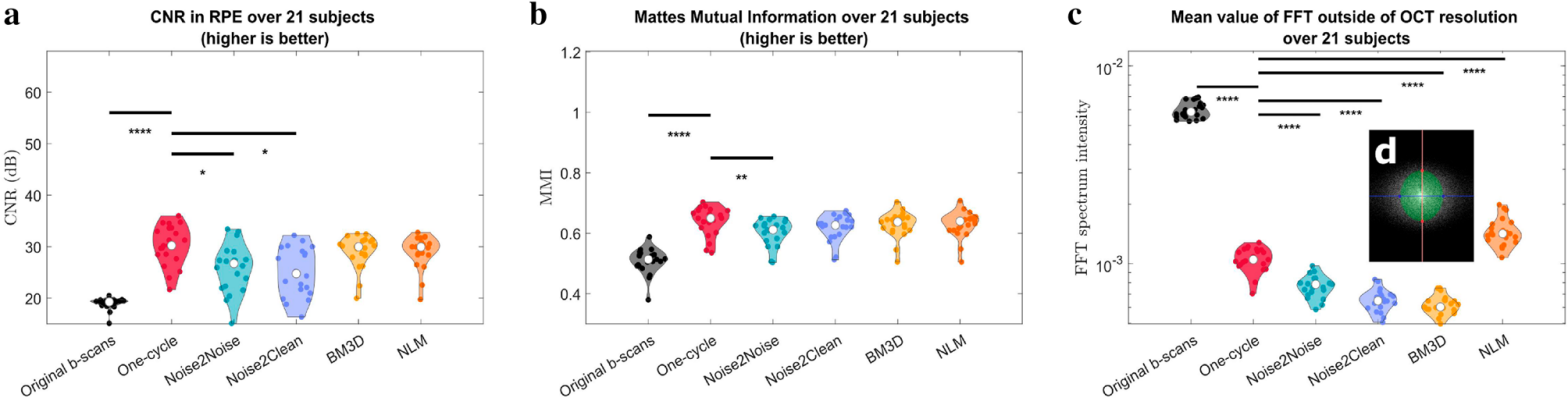
Image quality after denoising characterized by three different global metrics. All metrics were computed in the original B-scans (black violin plots) and for the five denoising methods studied (one-cycle workflow in red, N2N in turquoise, N2C in lavender, BM3D in yellow and NLM in orange). The Wilcoxon test was applied between the one-cycle workflow metrics and every other method (including no denoising, i.e., the original B-scans). Significant differences between methods are indicated by horizontal bars; asterisks indicate statistical strength. (**A**) CNR computed in the RPE. (**B**) MMI computed in whole images. (**C**) Mean intensity of the FFT spectrum beyond theoretical OCT resolution (**D**). (**D**) Ellipse (green) constructed using the axial and transverse OCT resolutions for the values of the half axes (pink and blue dots). The intensities of the frequency spectrum are averaged outside this ellipse.

We measured the contrast and speckle noise in the images by calculating the CNR in the RPE, and the preservation of image information by evaluating the Mattes Mutual Information (MMI) on whole B-scans. Noise reduction was also quantified by analyzing the intensity of the 2-dimensional Fast Fourier Transform (FFT) outside of an ellipse representing the resolution limit of our OCT machine (Fig. 2); no useful information can be contained in the spatial frequency spectrum beyond the resolution of the images. We found that the SNR and CNR computed in the choroid, RNFL and RPE regions yielded quite similar results; thus, only the CNR is discussed further, but both metrics are presented in the Supplementary Fig. A1.

Figure 2 shows the denoising performance as measured by the global image metrics. In Fig. 2A, all five denoising methods show similar CNR. A significant difference was found between the original B-scans and the one-cycle workflow, but none was found between the one-cycle method and any other denoising method. (The Wilcoxon test results are seen at the top the graphs: the horizontal bars and asterisks indicate differences between pairs of methods and their statistical strength). There is a fold-change increase in CNR of 1.57, 1.41, 1.31, 1.56 and 1.55 between the original B-scans and the one-cycle, N2N, N2C, BM3D and NLM denoising methods respectively (fold changes were computed between the median values, with the methods always in the same order: one-cycle, N2N, N2C, BM3D and NLM). The MMI (Fig. 2B) increases by fold changes of 1.27, 1.19, 1.22, 1.24 and 1.25. While the trend is analogous to the CNR, a smaller but significant difference was found between the one-cycle and N2N denoised images. The high frequency components outside the theoretical limits of the OCT resolution display a significant reduction after denoising (fold change decreases of 5.59, 7.46, 9.03, 9.71 and 4.13). All methods showed reduction of signal outside the OCT resolution; a significant decrease was also found between the one-cycle denoised images and each of the denoising methods. Note however that the NLM method doesn’t achieve the same reduction with a fold change of only 4.13 compared to other methods, indicating that a higher quantity of high-frequency noise persists after denoising.

**Figure 3:**
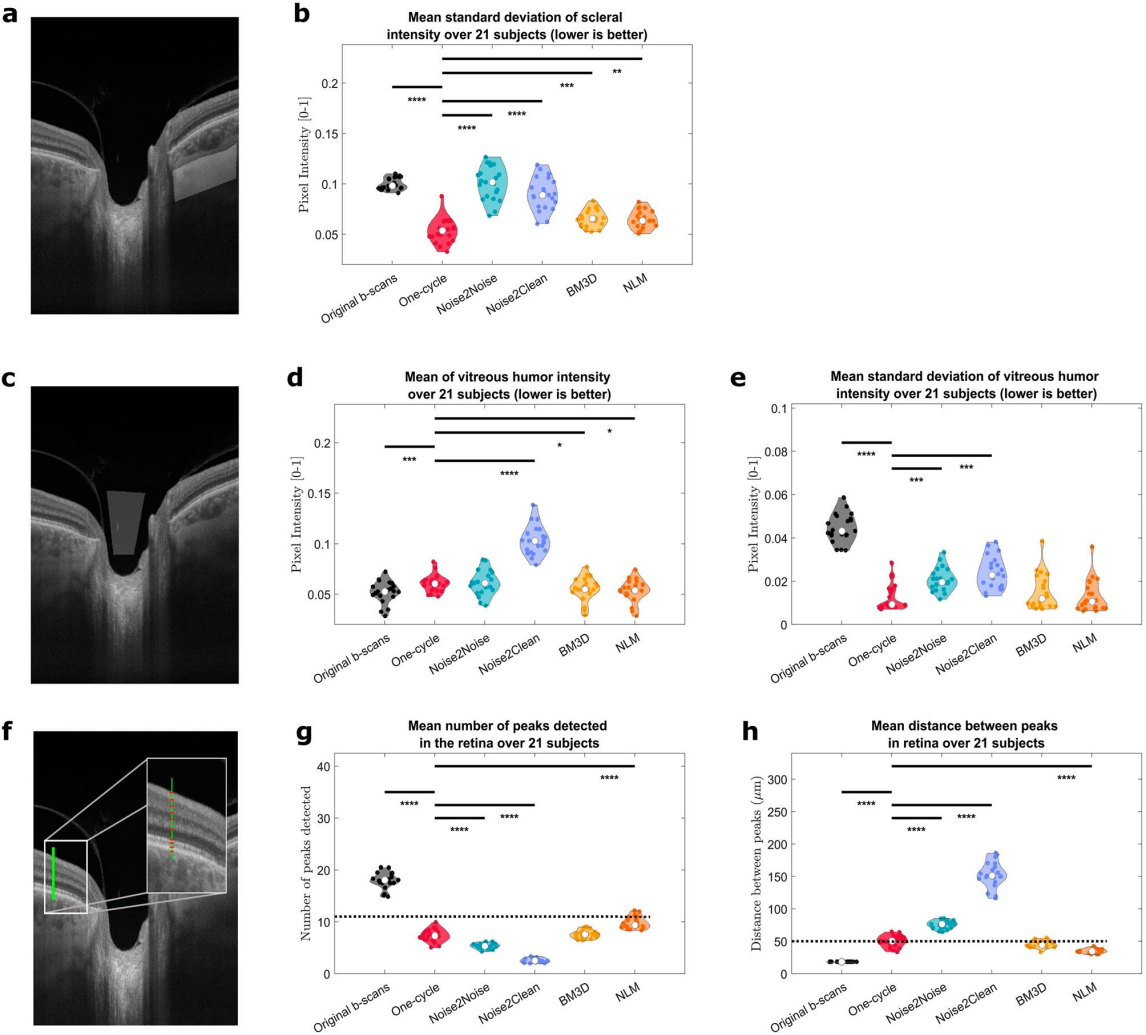
Region-specific assessment of denoising using five different metrics. All metrics were computed in the original B-scans (black violin plots) and the five denoising methods studied (one-cycle workflow in red, N2N in turquoise, N2C in lavender, BM3D in yellow and NLM in orange). The Wilcoxon test was applied between the one-cycle workflow metrics and every other method (including no denoising, i.e., the original B-scans). Significant differences between methods are indicated by horizontal bars; asterisks indicate statistical strength. Horizontal dotted lines show the expected values of the metrics when applicable. (**A**,**C**,**F**) Region considered to compute the metrics and an example. (**A**,**B**) Variation of pixel intensity in the sclera (expected value = 0); (**C**–**E**) metrics in the vitreous humor (expected mean and variation of 0); (**F**–**H**) number of retinal layers detected and average thickness (ideally 11 detected layers with a mean thickness of 50 µm).

The CNR and MMI both increase significantly using all five denoising techniques, indicating improved contrast and highest similarity between the scans after denoising. The signal present in the FFT spectrum outside the theoretical OCT resolution decreases for every denoising technique, which is consistent with reduced speckle noise. However, there is also a significant decrease in that metric between the one-cycle, N2N, N2C and BM3D denoised images close to the resolution limit. This highlights the blurring of the images by both neural networks and BM3D method (visible also in Fig. 1). Thus, while the speckle noise is reduced, denoising produced by those strategies induces a loss of real spatial information, which can also be seen in the retina-specific metrics (Fig. 3G,H). These three global image quality metrics assess the overall quality improvement of the denoised images by all five methods but are not able to discriminate between them or explain certain effects remarked on specific regions of the scans.

### Region-specific metrics: retinal biomarkers are blurred by CNN-based denoising strategies

Figure 3 shows the results of comparing the denoising methods based on region-specific metrics. The standard variation computed in the sclera, expected to be close to zero (Fig. 3B), clearly decreases between the original B-scans and all denoising strategies. While the trend is similar between the N2C and one-cycle denoised images, a significant difference is found between the one-cycle images and all other denoised images. The fold change decreases for this metric are of 1.83, 0.96 (increase of 1.04), 1.10, 1.50 and 1.55.

Both the mean and standard deviation of the pixel intensities in the vitreous humor should be close to zero (Fig. 3D,E). All denoising methods cause an increase in the mean pixel intensity values, with fold changes of 1.15, 1.16, 1.95, 1.04 and 1.02. The standard deviation is more discriminative between the denoising methods, with fold change decreases of 4.63, 2.20, 1.89, 3.61 and 4.06.

We assumed the retina is composed of 11 layers with an average thickness of approximately 50 µm. This metric is highly discriminative between denoising methods, with median values of 7, 5, 3, 8 and 9 layers detected (fold change decrease of 2.46, 3.37, 7.04, 2.38 and 1.93) and 51, 77, 151, 45 and 35 µm (fold change increase of 2.77, 4.20, 8.28, 2.44 and 1.92) for their respective average thickness (Fig. 3G,H).

Ocular structures of homogenous tissue characteristics (sclera and vitreous humor) are denoised by all strategies. The one-cycle workflow produces images with small standard deviations, nevertheless increasing the average pixel intensity in the vitreous humor compared to raw data (Fig. 3D,E). The performance of the N2N network is relatively close to the one-cycle workflow, however this network shows significant increases in the mean pixel intensity in the sclera as well as in standard variation in the vitreous humor compared to the proposed method. While the variation in the sclera is not significantly different between the one-cycle and N2C-denoised images, the performance in the vitreous humor, both for mean intensity and standard deviation, is significantly worse for the supervised network. The BM3D and NLM methods achieve slightly better performance in terms of standard deviation of pixel intensities in the sclera and vitreous humor.

Distinguishing the layered structure of the retina, a key clinical landmark, is best achieved with the non-AI denoising strategies. A lower-than-expected number of layers is found for the N2N model, and an abnormally low number of layers for the N2C model. While the extracted number of layers for the one-cycle denoised images is higher, it still falls short of the expected value. This is mainly due to the low contrast between certain layers (e.g., the ganglion cell and inner plexiform layers) that are often detected as one homogeneous layer, even in highly contrasted denoised images. This phenomenon is noticed for the BM3D denoising method, while the layer detection in the NLM-denoised scans is closest to the expected values. We observe the same trend for the mean distance detected between layers. While the one-cycle method yields an average of 51 µm between layers, BM3D of 45 µm, NLM of 35 µm (lower than expected) and the N2N method a value of 77 µm (higher than expected), the N2C method produces an abnormal mean distance of 151 µm. This is caused by the excessive blurring induced by the AI strategies, resulting in fewer peaks with larger distances between them; in the noisy original scans, the opposite situation yields many peaks and smaller distances.

### Movement preservation: AI, BM3D and NLM denoising strategies hide physiologically induced movement in scans

**Figure 4:**
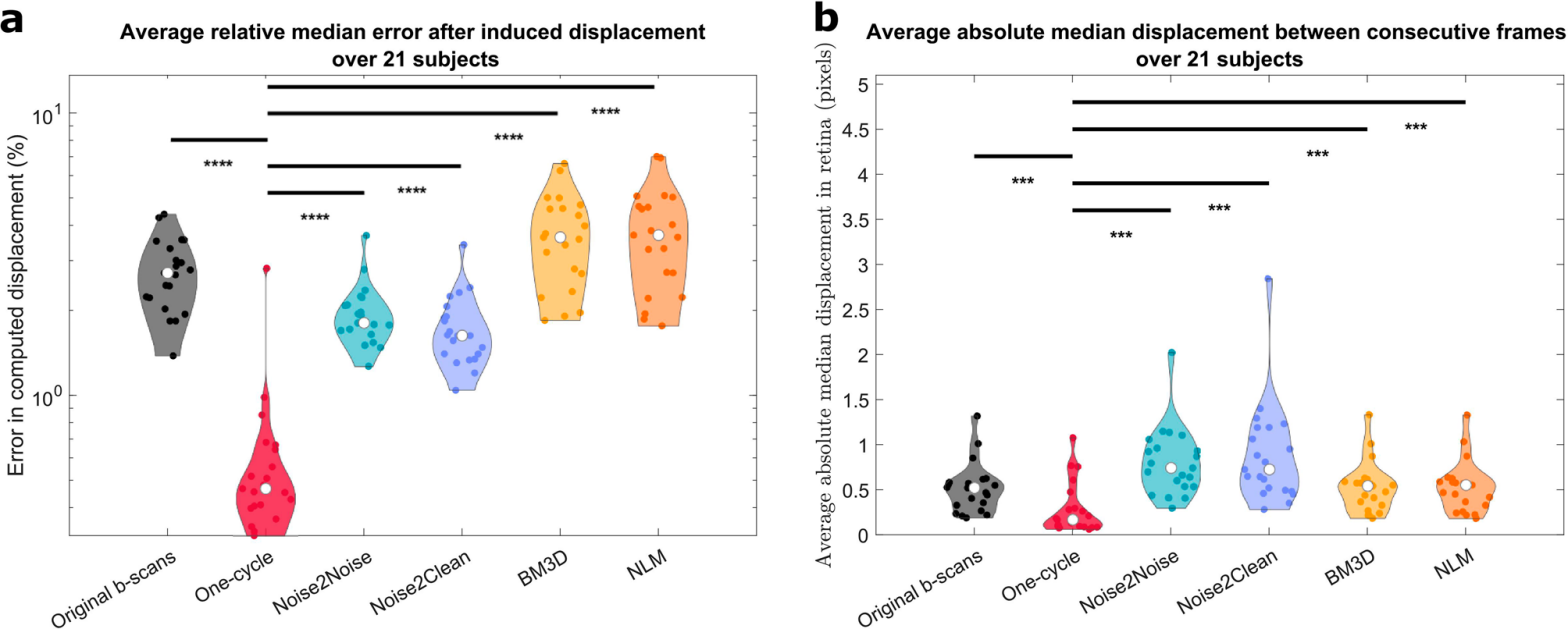
Movement preservation after denoising with the five different strategies. (**A**) The average absolute error between an induced and a computed displacement field was calculated for 100 pairs of images. The metric was computed in the original B-scans (black violin plots) and the five denoising methods studied (one-cycle workflow in red, N2N in turquoise, N2C in lavender, BM3D in yellow and NLM in orange). The Wilcoxon test was applied between the one-cycle workflow metrics and every other method (including no denoising, i.e., the original B-scans). Significant differences between methods are indicated by horizontal bars; asterisks indicate statistical strength. (**B)** The average absolute median displacement was computed between consecutive frames after denoising, for 100 pairs of denoised images by each strategy.

Denoising strategies must preserve true intensities of the scans, the anatomy of tissues and, in temporal acquisitions, allow quantification of movement of these tissues. In order to benchmark this capacity, we calculated the tissue displacement fields from artificially deformed images, and using consecutive frames of a movie acquired only 10 ms apart. Figure 4A shows the average median relative error after inducing a Gaussian expansion of the choroid of 5 pixels of amplitude and 20 pixels of standard deviation on consecutive pairs of images after denoising. This deformation metric illustrates fold changes between every metric and the original B-scans cohort of 3.69, 1.13 and 1.24 decreases for the one-cycle, N2N and N2C methods respectively, and of 1.35 and 1.35 increases for BM3D and NLM. The one-cycle method outshines the other denoising methods in preserving an induced deformation in pairs of images. The neural networks perform slightly better than the noisy scans, while the traditional methods seem to modify the induced deformation (if they erase, amplify, or misdirect the deformation was not studied). Figure 4B assesses the displacement present between pairs of consecutive frames after denoising by all strategies. Consecutive scans are obtained at 100 Hz and, as such, should contain small but non-null tissue deformation. Figure 4B shows an average median displacement in the order of a pixel for every denoising strategy.

## Discussion

All five methods achieve a significant reduction of speckle noise, but a closer look at the results reveals important differences. Overall, the one-cycle workflow produces the highest quality scans, in terms of speckle noise reduction, definition of ocular structures and preservation of movement. Figure 1 shows that the N2C model increases the overall intensities of the scans, a trend corroborated in Fig. 3D, while the N2N model enhances the contrast of different structures. Figure 4 shows that BM3D and NLM strategies, while obtaining good results in terms of region-specific metrics (Fig. 3), are not reliable regarding the accuracy of movement quantification. Whether this phenomenon comes from induced artefacts in the denoised images needs to be further investigated.

Overall, the one-cycle denoising presents several advantages compared to the other alternatives. First, our method is simple and effective in the context of dynamic OCT acquisition. It is easy to implement and requires only the addition of a pulse sensor connected to the subject’s finger during the video acquisition. The computational workload is quite low, and the code needed to synchronize the pulse signal and OCT timestamps can be adapted to clinical OCT equipment. Most importantly, the rationale for the algorithm is rooted in basic physiology and hence clear and understandable. The result follows a well-known method, and the output can be easily comprehended, and even debugged, for clinical decisions.

Denoising methods requiring pairs of images, such as our one-cycle method and both neural networks approaches, depend heavily on the accuracy of the eye tracking system of our OCT machine. This system’s precision is an ongoing challenge in both commercial and research available OCT apparatus. Averaging images corresponding to different ocular structures, or trying to transform one ocular plane into another, would result in overall bad behavior of those three methods. Those same approaches depend on the accuracy of the image registration as well. The global registration removes large changes due to breathing, head and eye movement, while the finer A-scan registration corrects tilt motion artefacts that occur during a B-scan acquisition and fixation issues. These tilting artefacts are typically not corrected by common rigid registration techniques, nor by the hardware-based motion artefact correction modules of commercial OCT machines. For videos spanning long periods (30 s in our case), A-scan tilts can accumulate over time and severely hinder the registration and matching of structures between pairs of images. Left uncorrected, they lead to blurring artefacts in ocular structures when averaging several images; they can also bias the reconstruction of denoised scans in CNN approaches. The cross-correlation A-scan registration technique we use is an efficient and simple method to correct these tilts.

It is important to note that what renders the one-cycle method efficient can also be considered a shortcoming: as it assumes temporal redundancy between cardiac cycles, any non-periodic information in the video will be lost. An approach not relying on averaging between cycles could, in principle, detect abnormalities manifesting a non-periodic behavior. Alternatively, averaging to several cycles rather than a single one is possible, but the quality of the denoised images would be affected. In order to reach the same level of image enhancement as one-cycle, the OCT acquisition time would need to be increased proportionally. This could lead to difficulty in registering the images, for example due to increased subject movement caused by fatigue.

In comparison, the N2N workflow can train a network in an unsupervised way that still gives high quality results. Once trained, the model only needs the current image to output a denoised version, and temporal information from multiple heart cycles is preserved. However, the reliability of the denoised scans at the pixel level is not assured, even if in the present case, only simple convolution and pooling operations are used. In order to use the AI-denoised images for biomarker quantification (pixel-wise), more work would need to be done in understanding how the network’s operations affect its reliability. This is true for BM3D and NLM as well, being single scans denoising methods which obtain high image quality metrics, but where the trustworthiness of the pixel intensities is not guaranteed. In contrast, the one-cycle method establishes a reliability that is lacking in these other automated approaches.

### Limitations of the study

The Noise2Noise (and Noise2Clean) workflows were used in this work with a simple U-net architecture. This training scheme and architecture could potentially be improved, as has been tried in the past (e.g., GANs or fully connected convolutional networks) in the OCT denoising field. A more complete comparison between image-processing-based and neural network denoising methods could be carried out with more complex models as well (e.g., vision transformers). However, these architectures come with increased complexity of data acquisition and training. Moreover, the reliability of denoised pixel intensities would need to be extensively assessed, especially in the case of generative methods.

## Conclusion

The proposed one-cycle workflow is a simple and effective denoising method to facilitate analysis of dynamic OCT data. The temporal redundancy of single-scan video acquisitions allows to average scans without the risk of blurring small movements needed for dynamic analysis of the tissues, as is risky with classical averaging techniques. Compared to traditional denoising strategies such as BM3D and NLM in addition to convolutional neural network (CNN) architectures in both unsupervised and supervised training schemes, our one-cycle method achieves the highest denoising performance, increases image quality globally and in specific areas of the eye, and preserves physiological motion before and after denoising. In comparison, CNNs, particularly in unsupervised training, achieve remarkable performance but blur the images. Hence, they achieve high denoising performance in terms of CNR and MMI, but at the cost of losing high-resolution details of ocular structures. BM3D and NLM strategies are easy to use and quantitatively improve image quality, but do not preserve induced deformation. While the one-cycle method benefits from multiple input images, all other denoising techniques are single-scan methods and thus achieve impressive performance.

## Methods

### One-cardiac-cycle OCT videos

We present a denoising strategy based on the assumption that ocular tissue images in a temporal sequence will repeat periodically with the heart cycle. Images from the same cardiac phase can thus be averaged without blurring changes due to movement or physiological processes. Rigid registrations applied to the B-scans in a video are sufficient to correct global movement due to eye saccades, lack of gaze fixation and breathing. The resulting video captures changes due only to the blood flow in and out of tissues. As such, the registered frames corresponding to the same instant in a cardiac cycle, i.e., all frames taken at equal delays with respect to systole, should look the same. Averaging only those specific images reduces speckle and other noise sources present in the images while preserving visible pulsation-driven movements.

**Figure 5:**
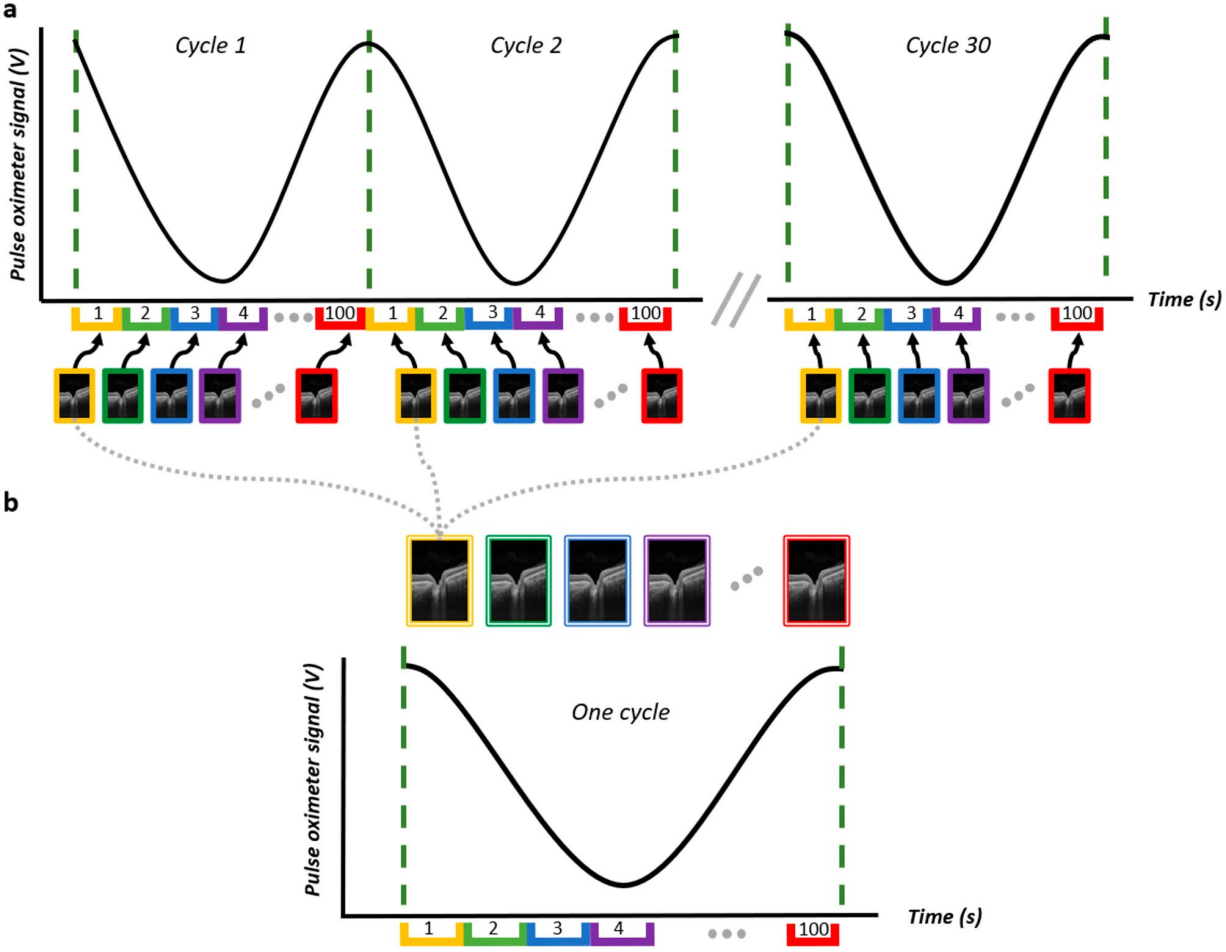
Visual representation of the one-cycle workflow. (**A**) OCT B-scans are assigned to time bins according to the correspondence between their timestamp and the pulse signal. (**B)** All frames assigned to the same bin are registered and averaged to create the one-cycle images with reduced speckle noise.

We acquire OCT videos at a B-scan rate of 100 Hz for a duration of approximately 30 s (see section “Data acquisition”). These videos image a single plane of the retina and optic nerve head throughout multiple heart cycles. The heart cycle is simultaneously measured with a pulse sensor and digitized. The peaks of the cardiac signal are detected, and the period is divided into 100 intervals, referred hereafter as *bins*. Images are assigned to bins using the timestamps that indicate the moment of acquisition of every frame of the OCT movie. The 3000 frames of our movies are thereby split in 100 bins; each bin receives approximately 30 frames representing identical instants of different cycles. For every bin, the registration steps described in section “Image registration” are applied, using the first frame of that bin as the reference frame. After registration, all images in every bin are averaged together, yielding a single denoised image. To align the averaged frames together and recreate a spatially coherent movie, a second registration is applied, using the same workflow as in section “Image registration”. This transforms a 30-s-long OCT video of 3000 frames into a one-cycle, approximately 1-s-long video, where every frame is the average of approximately 30 images. See Fig. 5 for a visual representation of this workflow.

### Data acquisition

Time series are obtained using the Zeiss PlexElite 9000, a swept-source OCT machine. We use a custom mode to acquire 30-s-long videos at a B-scan frame rate of approximately 100 Hz and an A-scan rate of approximately 100 kHz. An eye tracker ensures that the same region of the eye is being imaged throughout the whole 30 s. This set-up produces 3000-frames B-scan movies mapping a single plane of the optic nerve head (ONH) in less than a minute. The output images have a size of 1536 × 1024 pixels, with pixel size of 1.95 µm axially and approximately 5.9 µm transversally. The OCT resolution is of 6.3 µm axially and 20 µm transversally. A more detailed description of the data acquisition protocol was previously published^12^.

The subject’s heart beats are measured simultaneously with the OCT video using a pulse sensor (PulseSensor, World Famous Electronics llc.) in contact with the subject’s finger. This sensor shines light (λ = 515 ± 15 nm) on the finger and a photodiode measures the backscattering intensity, which is proportional to oxygenated hemoglobin. Signal digitization is achieved with an analog-to-digital-converter (ADS1263, Waveshare) connected to a Raspberry Pi (Model 4B). This set-up was first described elsewhere^39^. 21 videos of different eyes were analyzed in total, corresponding to 15 different subjects (some were imaged in both eyes).

### Image registration

We use a two-step registration workflow: a global translation using an arbitrary reference frame (described in section “One-cardiac-cycle OCT videos”), followed by a fine A-scan alignment. The latter step serves to correct a progressive rotational misalignment (tilt) that can affect the individual A-scans. We correct the A-scan tilt by calculating the correlation between pairs of A-scans from the reference and moving images. The maximum correlation between pairs of A-scans is the axial shift that could be applied to the moving A-scan to optimally register it to the reference image. This shift is calculated for all pairs of A-scans and a linear fit is obtained. Axial shifts are applied based on the linear fits computed for every image. In the interest of speed, correlations are only computed every 20 A-scans.

An outlier detection scheme is used to detect frames where the registration process would not have worked. The Mattes Mutual Information (MMI)^40^ metric is computed between all frames corresponding to the same cardiac phase, where the first frame of each bin is taken as the reference. This similarity metric allows to detect images where the registration would have diverged, resulting in artifacts. Otsu thresholding is used to split the whole set of MMIs computed on every frame of the movie, after which a binomial distribution is fitted on the split. Goodness of fit indicates whether frames can be detected as outliers as such the lower MMIs corresponding frames are removed.

### Global image quality quantification

Different metrics are used to quantify image quality before and after denoising has been applied.

We use Mattes Mutual Information (MMI)^40^ to compute the similarity between whole image pairs before and after denoising:

Mattes Mutual Information:

1 $$MMI= \sum_{x \in X}\sum_{y \in Y}{P}_{\left(X, Y\right)}\left(x, y\right){\text{log}}\left(\frac{{P}_{\left(X, Y\right)}\left(x, y\right)}{{P}_{X}\left(x\right){P}_{Y}\left(y\right)}\right)$$

where *P* represents the probability distribution of pixels in an image, and *x* and *y* are pixel intensity values of images *X* and *Y*.

We use the signal-to-noise ratio (SNR) (Eq. (2)) and the contrast-to-noise ratio (CNR) (Eq. (3)) to quantify the noise present in structures of interest:

Signal-to-noise Ratio:

2 $${SNR}_{dB}=20 \cdot {{\text{log}}}_{10}\left(\frac{{\mu }_{i}}{{\sigma }_{b}}\right)$$

Contrast-to-noise Ratio:

3 $${CNR}_{dB}=20 \cdot {{\text{log}}}_{10}\left(\frac{|{\mu }_{i}-{\mu }_{b}|}{{\sigma }_{b}}\right)$$

where $\mu$ and $\sigma$ are the mean and the standard deviation of the intensity, respectively, and index *i* refers to a region of interest (ROI) and *b* to the background. SNR and CNR are calculated in manually segmented ROIs corresponding to the choroid, retinal nerve fiber layer (RNFL) and retinal pigment epithelium (RPE). ROIs were identified on one B-scan for every video by the same observer. The ROI corresponding to the background was located in the bottom portion of the B-scan on both sides of the ONH.

We use the 2-dimensional Fast Fourier Transform (FFT) to compute the signal content of the images in the frequency domain. Based on the specifications that determine axial and transverse resolutions of the OCT machine, we define an ellipse that contains useful information. We calculated the semi-axes of this ellipse at the cut-off frequencies corresponding to the axial and transverse resolution of the OCT machine. Signal outside this ellipse necessarily corresponds to noise, thus the average spectral intensity in that region measures the overall noise level. See Fig. 2D for a visual representation of the FFT spectrum and the ellipse.

### Region-specific image quality quantification

To allow more meaningful comparisons between different denoising strategies, we designed several metrics to describe the image clarity of different ocular structures. ROI-specific metrics are defined and computed for the sclera, vitreous humor and retinal layers. All ROIs are manually segmented on one frame in each video by the same observer.

At the resolution provided by an OCT, the *sclera* is a homogeneous collagenous layer. Thus, variation of intensities in this region should be minimal; the standard deviation is computed and expected to be close to zero.

As the OCT signal in the *vitreous humor* should be null, the mean and the standard deviation of the pixel intensities are computed in this region, and both are expected to be close to zero.

The different *retinal layers* are expected to be clearly defined and distinguishable. Starting from the inner limiting membrane and ending with the Bruch’s membrane, we count 11 interfaces that should be found in denoised OCT scans. These interfaces include the transitions between the retinal nerve fiber layer, ganglion cell layer, inner plexiform layer, inner nuclear layer, outer plexiform layer, outer nuclear layer, external limiting membrane (very thin layer that would be counted as one delimitation), photoreceptor inner and outer segments (that are visibly divided into three sub-layers) and finally the retinal pigment epithelium. The thicknesses of these layers vary along the retina and depend on the exact position of the scan, as well as on inter-subject variability. We considered as optimal an average thickness of approximately 50 µm^41^. To measure this, we extract the locations and the distances between peaks of the vertical gradient across the retina, from the RNFL to the RPE (Fig. 3F). An average of the number of detected layers and their average thickness was computed over all lines extracted in the ROI.

### Movement preservation quantification

A good denoising strategy removes noise while preserving tissue anatomy. Furthermore, using temporal data allows the analysis of the dynamics and biomechanical properties of tissues. As such, we designed a metric assessing whether physiological movement induced in scans was still visible after denoising.

We followed a methodology we have used in the past^12^: a known displacement field simulating the expansion of the choroid was created. 100 pairs of consecutive scans in the original and the one-cycle videos were identified, after which the second scan of each pair was warped with the known deformation field (for the one-cycle videos, those hundred scans were simply the one-cycle video itself). All pairs of scans were denoised using their respective denoising strategies, after which the displacement field was computed again between both denoised scans with the demons algorithm^42,43^. The average absolute median error was computed between the known and computed displacement fields in the retina.

We also quantified the displacement occurring between pairs of consecutive frames after denoising (without inducing artificial deformation). The average median displacement was computed in the retina over 100 pairs of images for each denoising strategy.

### BM3D and NLM denoising approaches

The Non-Local Means (NLM) approach denoises images based on averaging neighborhood pixels based on their similarity. The similarity is computed using the Euclidean distance between the pixel values. We used the MATLAB implementation of the algorithm provided by the authors^38^.

The Block-matching and 3D filtering (BM3D) algorithm^37^ was originally inspired by the NLM approach, and is based on the Wiener filtering of similar groupings of pixels called blocks. The MATLAB implementation of the algorithm published by the authors was used.

### Neural networks implementation

We implemented supervised and unsupervised training schemes for the Noise2Noise architecture. The model architecture, training sets, hyperparameters and metrics used are described in the following subsections.

#### Noise2Noise

The Noise2Noise (N2N) neural network framework denoises images in an unsupervised manner. The method is based on training the network using pairs of images representing the same structures with different noise levels. In the original article^25^, synthetic noise was added to the images to simulate these pairs.

Using the same assumption as for the one-cycle videos, we suppose that registered frames corresponding to the same instant in the cardiac cycle represent the same structures but with different noise distributions. Hence, frames detected to be in the same temporal bin are considered as pairs and used as input and output of the N2N framework (registration is described in section “Image registration”). All combinations of these pairs of frames are used as the N2N training set. Images are cropped to facilitate training to a fixed size of 512 by 512. Cropped patches are located randomly in the images, 20 patches are extracted from each image to introduce variation to the training set, and a total of 16,812,180 patches are used. We use a U-net-like architecture as the network backbone^44^. The convolutional blocks are composed of two convolutional layers as well as a max pooling (resp. upsampling) layer on the encoder (resp. decoder) pathway. Skip connections are used, as in the original U-net. No drop-out is used. The ReLU activation is used whenever applicable. We choose the Adam optimizer with ${\beta }_{1}=0.9, {\beta }_{2}=0.99, \varepsilon ={10}^{-8}$.

#### Noise2Clean implementation

As in other works that tested N2N, we compared the performance of the unsupervised framework to a supervised counterpart, which we named Noise2Clean (N2C). The N2C model architecture and gradient descent optimizer are the same as in the N2N workflow, the difference lying in the training dataset. In N2C, the network is trained to transform a noisy B-scan into its corresponding one-cycle averaged frame. As such, N2C corresponds to a supervised training scheme where the inputs are the noisy frames and the outputs are the corresponding denoised frames, using the one-cycle workflow to provide the ground truth samples. All combinations of these pairs of frames are used to compose the N2C training set. The training set comprises a total of 1,196,480 patches.

#### Training

We use a batch size of 4 cropped images. The total image sets are split into training, validation, and testing, with proportions 64%, 16% and 20%, respectively. We choose a grid search approach to vary several parameters such as the number of convolutional blocks used in the U-net-like architecture, the number of features present at each convolutional layer, the starting learning rate and the loss function used. Monitoring of the training uses the weights and biases library. The model architectures and training were implemented using the Keras library in Python. A fixed number of 300 epochs is used for training, with early stopping to end the training when the validation loss stops improving. The same training sets are used for all combinations in the grid search.

Four different loss functions are tested: mean squared error (MSE), mean absolute error (MAE), cosine similarity function and L_0_ error function, as defined elsewhere^25^. We use the peak signal-to-noise ratio (PSNR) and Structural Similarity Index Metric (SSIM)^45^ (Eqs. (4) and (5)) to evaluate the performance of the models. Those that achieve the highest validation SSIM and PSNR while limiting the presence of artefacts are chosen as best.

Peak signal-to-noise ratio (PSNR):

4 $$PSNR=10 \cdot {{\text{log}}}_{10}\frac{{MAX}_{I}^{2}}{MSE}$$

Structural Similarity Index Metric (SSIM):

5 $$SSIM=l\left(x, y\right)\cdot c\left(x, y\right)\cdot s(x, y)$$

The final N2N model consists of a U-net architecture made up of 6 convolutional blocks in each pathway, using a starting number of 64 filters per convolutional layer, and training with an initial learning rate of 10^–5^. The final N2C model consists of the same optimal hyperparameters, apart from the starting learning rate which is of 10^–4^.

### Ethical compliance

Human subjects were included in this study. This study was approved by the institutional review board of Maisonneuve-Rosemont Hospital and was performed in accordance with the 1964 Declaration of Helsinki and its amendments. Written informed consent was obtained from all participants. No animal subjects were used in this study.

### Supplementary Information

Supplementary Legends.

Supplementary Information 1.

Supplementary Video 1.

Supplementary Video 2.

Supplementary Video 3.

Supplementary Video 4.

Supplementary Video 5.

Supplementary Video 6.

Supplementary Video 7.

Supplementary Video 8.

Supplementary Video 9.

Supplementary Video 10.

Supplementary Video 11.

Supplementary Video 12.

## Data Availability

The datasets used and analyzed during the current study will be available from the corresponding author on reasonable request.

## Code availability

The MATLAB code used to generate the one-cycle videos is available at https://github.com/EmmanuelleRicher/One-cycle-denoising.
