# Supplementary material for: Denoising OCT videos based on temporal redundancy
Source: Sci Rep. 2024 Mar 19;14:6605. doi: 10.1038/s41598-024-56935-0 (PMC10951312; doi:10.1038/s41598-024-56935-0)
Supplement: Supplementary file 1 — Supplementary Legends. [file 41598_2024_56935_MOESM1_ESM.docx]

Supplementary Materials

Denoising OCT videos based on temporal redundancy – Supplementary materials

Emmanuelle Richer^1, 2^, Marissé Masís Solano^2, 3^, Farida Cheriet^1^, Mark R. Lesk^2, 3^, Santiago Costantino^2, 3, *^

1: Department of Computer Engineering and Software Engineering; École Polytechnique de Montréal; Montreal, Quebec, H3T 1J4; Canada

2: Maisonneuve-Rosemont Hospital Research Center; Montreal, Quebec, H1T 2M4; Canada

3: Department of Ophthalmology; Université de Montréal; Montreal, Quebec, H3T 1P1; Canada

*: Corresponding author: santiago.costantino@umontreal.ca

*Appendix B: supplementary videos and legends*

The one cycle method and trained networks were applied on new test data, never seen before during the training process. Videos in supplementary material show the results of every denoising method on two different subjects. Following is the list of provided videos and their short descriptions.

*supplementary_video_1.mp4*: Original B-scans without any denoising or registration of subject 1

*supplementary_video_2.mp4*: One-cycle video repeated 10 times for visualization purposes of subject 1

*supplementary_video_3.mp4*: Video of subject 1 after rigid registration and denoising with N2N network

*supplementary_video_4.mp4*: Video of subject 1 after rigid registration and denoising with N2C network

*supplementary_video_5.mp4*: Video of subject 1 after rigid registration and denoising with BM3D

*supplementary_video_6.mp4*: Video of subject 1 after rigid registration and denoising with NLM

*supplementary_video _7.mp4*: Original B-scans without any denoising or registration of subject 2

*supplementary_video _8.mp4*: One-cycle video repeated 10 times for visualization purposes of subject 2

*supplementary_video _9.mp4*: Video of subject 2 after rigid registration and denoising with N2N network

*supplementary_video _10.mp4*: Video of subject 2 after rigid registration and denoising with N2C network

*supplementary_video_11.mp4*: Video of subject 2 after rigid registration and denoising with BM3D

*supplementary_video_12.mp4*: Video of subject 2 after rigid registration and denoising with NLM
