# Supplementary material for: Denoising OCT videos based on temporal redundancy
Source: Sci Rep. 2024 Mar 19;14:6605. doi: 10.1038/s41598-024-56935-0 (PMC10951312; doi:10.1038/s41598-024-56935-0)
Supplement: Supplementary file 2 — Supplementary Information 1. [file 41598_2024_56935_MOESM2_ESM.pdf]

Supplementary Materials

# Denoising OCT videos based on temporal redundancy – Supplementary materials

Emmanuelle Richer[1, 2], Marissé Masís Solano[2, 3], Farida Cheriet[1], Mark R. Lesk[2, 3], Santiago Costantino[2, 3, *]

1: Department of Computer Engineering and Software Engineering; École Polytechnique de Montréal; Montreal, Quebec, H3T 1J4; Canada

2: Maisonneuve-Rosemont Hospital Research Center; Montreal, Quebec, H1T 2M4; Canada

3: Department of Ophthalmology; Université de Montréal; Montreal, Quebec, H3T 1P1; Canada

*: Corresponding author: santiago.costantino@umontreal.ca

## *Appendix A: supplementary image quality metrics*

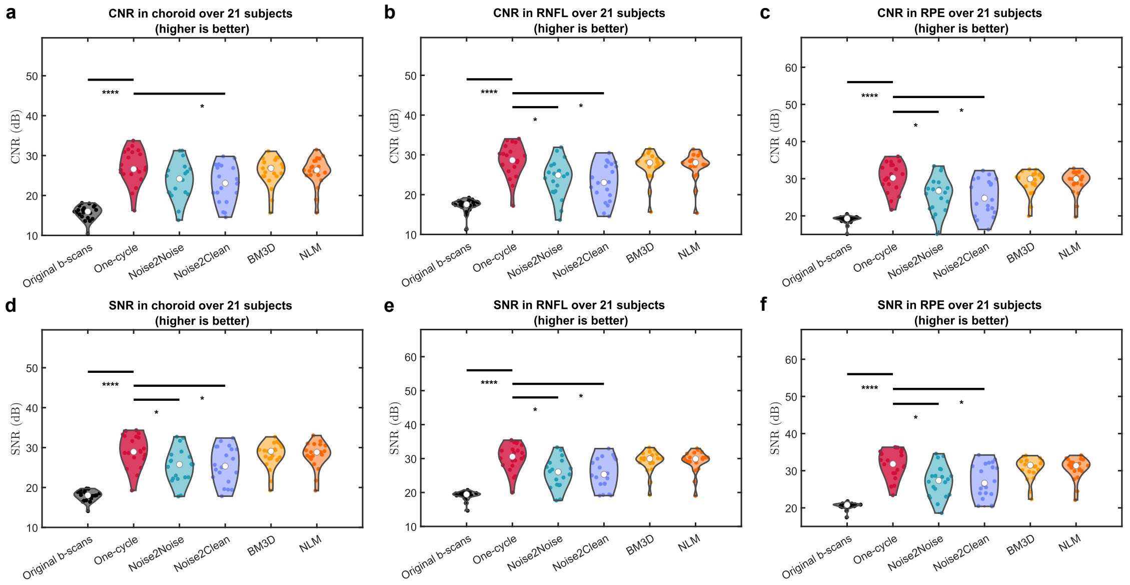


**CNR and SNR in the choroid, RNFL and RPE**

**Figure A.1**: SNR and CNR computed in the choroid, RNFL and RPE regions, according to the five denoising methods tested. Panels A, B and C show the CNR computed respectively in those three regions, while panels D, E and F show the CNR. For each distribution in the violin plots, datapoints are the average metric values for each eye, while the white dot is the median over all 21 eyes. The Wilcoxon test was applied between the one-cycle workflow and every other method (including no denoising, i.e., the original B-scans).

The fold change increases between the original B-scans and the five denoising methods (one-cycle, N2N, N2C, BM3D, NLM) are as follows: CNR in the choroid (1.66, 1.52, 1.45, 1.68, 1.65), CNR in the RNFL (1.63, 1.44, 1.32, 1.60, 1.60), CNR in the RPE (1.57, 1.41, 1.31, 1.56, 1.55), SNR in the choroid (1.60, 1.43, 1.42, 1.61, 1.60), SNR in the RNFL (1.57, 1.34, 1.32, 1.54, 1.54) and SNR in the RPE (1.53, 1.33, 1.31, 1.51, 1.50).
